# From Selenium- to Tellurium-Based Glass Optical Fibers for Infrared Spectroscopies

**DOI:** 10.3390/molecules18055373

**Published:** 2013-05-10

**Authors:** Shuo Cui, Radwan Chahal, Catherine Boussard-Plédel, Virginie Nazabal, Jean-Louis Doualan, Johann Troles, Jacques Lucas, Bruno Bureau

**Affiliations:** 1 UMR 6226 Sciences Chimiques de Rennes-Verres & Céramiques, Université de Rennes 1-CNRS, Campus de Beaulieu, 35042 Rennes Cedex, France; 2 Department of Materials Science and Engineering, Zhejiang University, Hangzhou 310027, China; 3 Centre de Recherche sur les Ions, les Matériaux et la Photonique (CIMAP), UMR 6252 CEA-CNRS-ENSICaen, Université de Caen, 14050 Caen, France

**Keywords:** selenide glass, telluride glass, optical fiber, infrared spectroscopy

## Abstract

Chalcogenide glasses are based on sulfur, selenium and tellurium elements, and have been studied for several decades regarding different applications. Among them, selenide glasses exhibit excellent infrared transmission in the 1 to 15 µm region. Due to their good thermo-mechanical properties, these glasses could be easily shaped into optical devices such as lenses and optical fibers. During the past decade of research, selenide glass fibers have been proved to be suitable for infrared sensing in an original spectroscopic method named Fiber Evanescent Wave Spectroscopy (FEWS). FEWS has provided very nice and promising results, for example for medical diagnosis. Then, some sophisticated fibers, also based on selenide glasses, were developed: rare-earth doped fibers and microstructured fibers. In parallel, the study of telluride glasses, which can have transmission up to 28 µm due to its atom heaviness, has been intensified thanks to the DARWIN mission led by the European Space Agency (ESA). The development of telluride glass fiber enables a successful observation of CO_2_ absorption band located around 15 µm. In this paper we review recent results obtained in the Glass and Ceramics Laboratory at Rennes on the development of selenide to telluride glass optical fibers, and their use for spectroscopy from the mid to the far infrared ranges.

## 1. Introduction

**Figure 1:**
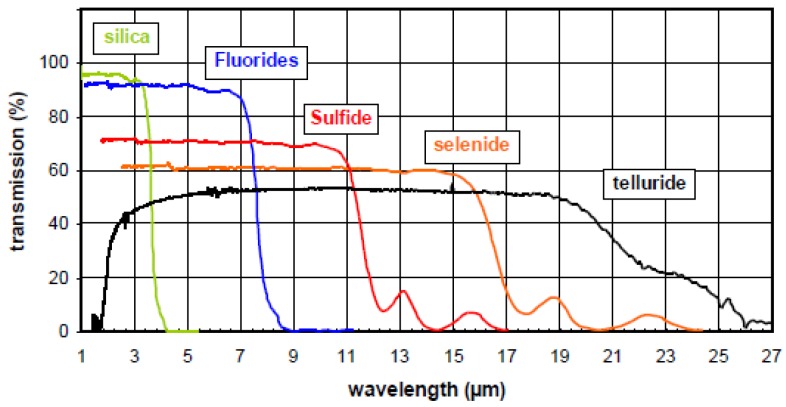
A comparison of the typical transmittance spectra of silica, fluorides, sulfide, selenide and telluride bulk glasses.

Chalcogenide glasses contain one or more chalcogen elements (e.g., sulfur, selenium or tellurium) and other additional elements such as arsenic, germanium, antimony, gallium, *etc*. During recent decades, research in this area has been primarily motivated by the optical properties of these glasses in the infrared region (Figure 1). Examples include infrared detectors, and moldable infrared optics such as lenses [1], infrared optical fibers [2] or planar waveguides [3].

Among them, selenide glasses are especially interesting due to their wide transparency in the mid-infrared region and favorable thermal stability. Based on these features, selenide glasses can be easily shaped into infrared optical devices such as optical fibers [4,5,6,7,8]. These fibers have been intensively studied for potential applications such as remote spectroscopy, laser power delivery, temperature sensing and thermal image transmission. In our group, these fibers have been under development for the past decade mainly to carry out Fiber Evanescent Wave Spectroscopy (FEWS) experiments [9]. As the transmission from 2 to 12 µm of selenide glass fiber corresponds to the fundamental absorption domain caused by vibrations of most of chemical and biological molecules, applications such as detection of molecules [10], or pollutants for environmental purposes [11] and follow-up of *in-situ* chemical reactions [12] could be explored by a close contact between fiber and specimen. Thanks to these fibers, some fruitful results were obtained, in particular in medical and environmental applications. At the same period, some double index single mode selenide glass fibers [13] were also fabricated, permitting one to observe and filter infrared signals at 10 µm.

In addition, due to their low phonon energy, selenide glasses with high refractive index and proper rare-earth (RE) solubility, could also be utilized to generate sources and lasers in the mid-infrared region [3]. Therefore, RE doped glass fibers were developed for fiber laser or optical amplifier applications in the mid-infrared range.

Moreover, another type of sophisticated selenide glass fiber was also developed: microstructured optical fiber [8] with its cross-section mostly covered by periodically arranged microstructures. This unique structure makes this kind of fiber suitable for gaseous sensing and endless single mode propagation.

Recently, tremendous interest has been focused on the exploration of the Universe. Thanks to the Darwin mission, which requires the development of a nulling interferometer operating in the IR for the detection of signatures of life on Earth-like planets, single mode selenide glass fibers, therefore, have been fabricated [13] to detect the signals of water vapor and ozone located at 6.2 and 9 µm, respectively. However, this is not a corroborating evidence of alien life outside the Solar System. Fibers enabling to observe the CO_2_ absorption band spreading around 15 µm should be developed. Tellurium-based glasses, due to their atoms’ heaviness, can transmit light further in the infrared region up to 28 µm [14]. Therefore, the telluride glass family has been revisited for the DARWIN mission led by the European Space Agency (ESA). New glass compositions only based on tellurium were discovered [14,15,16]. 

For both selenide and telluride fibers, a crucial difficulty in the fabrication of this type of fiber is how to carefully control the impurity levels of the starting products as well as of the final fibers. In order to get a preform with high purity and exact composition, purification of raw materials and synthesis based on the use of sealed silica tubes under vacuum are required [17]. Indeed, carbon and water are frequent contaminants and should be removed from the glass batch by a combination of static and dynamic distillations. During the drawing step, the contamination by water must also be avoided by using a dry drawing atmosphere. The contact with oxygen in air should also be avoided by Ar protection. The different process steps have to be conducted at optimized temperatures to avoid any crystallization phenomena of the glass in the bulk as well as on its surface.

On the other hand, Se and Te are totally opposite in terms of their ability to form glassy materials. Whereas Se is a very good glass former, telluride glass tends to crystallize easier due to the metallic behavior of Te [18]. Finally, as discussed previously, the elaboration of pure Te-based glass is a clear issue and the manufacture of optical fiber is very challenging. Nevertheless, this new class of optical devices is essential to open the access to the detection IR vibration beyond 12 µm.

In this paper we present a review on our recent results on the development of selenide to telluride glass optical fibers, and their use for spectroscopy in the mid- to the far-infrared ranges.

## 2. Selenide Single Index Glass Fibers for Infrared Spectroscopy

**Figure 2:**
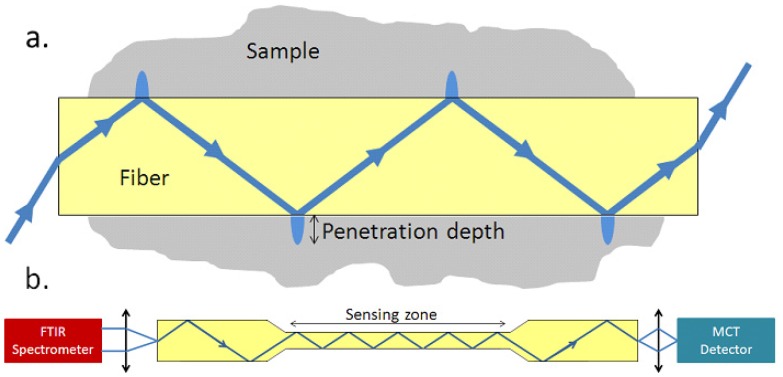
(**a**) Mechanism of fiber evanescent wave spectroscopy (FEWS); (**b**) General set up of FEWS and the scheme of a tapered fiber.

Since Compton [19] first reported chemical detection using chalcogenide glass fibers in 1988, this topic has been under investigation for more than 20 years. Up to now, selenide glasses have been revealed as good candidates for the elaboration of special fibers to be implemented in optical sensors. They are especially suitable for sensors based on an original spectroscopic method named Fiber Evanescent Wave Spectroscopy (FEWS), which uses the evanescent field formed as a beam propagates by total internal reflection at the interface between the waveguide and sample to test absorption peaks of samples at specific wavelengths (Figure 2a). 

The FEWS method is quite simple to implement since the measurement necessitates only a standard spectrometer equipped with special kits to focus the light and a MCT detector. Moreover, a large range of glass formulations is available to obtain suitable optical fibers with large infrared transparency ranges and low optical losses. Therefore, chalcogenide glass fibers for FEWS have drawn lots of attention in the last decades. More recently, selenide single index fiber-sensors have been successfully used and qualified, mainly for biomedical and environmental purposes, in the framework of multidisciplinary research programs [20,21].

For the FEWS technique, since a high refractive index could induce a deeper evanescent wave penetration depth and thereby increase fiber sensitivity, selenium-based glasses possess an inherent advantage. Besides, as fiber sensitivity could be greatly enhanced by reducing fiber diameter [22] or increasing contact length [23] between fiber and sample, we should be able to develop a Se-based fiber with small diameter and long contact length. Actually, in order to enhance sensor sensitivity, the diameter of sensing zone could be reduced by two routes [21]: etching [24] and reduction on-line by precise adjustment of fiber drawing speed [21]. The general set up of FEWS and the scheme of a tapered fiber are shown in Figure 2b.

The best glass composition, which affords the best compromise between infrared transparency and glass stability, is a Te-As-Se (TAS) glass system with about 50% Se [25]. Besides, other glass systems such as As_2_Se_3_ [10], GeSe_4_ [26] and Sb-Se-Ga-Ge [20] are also utilized in fiber sensing.

For Te_2_As_3_Se_5_ glass, some tapered fibers were successfully elaborated. The absorption peaks of C=O bond of acetone diluted in methylene chloride were tested and compared when the fiber diameter or contact length were changed [21].

However, the most important application of Se-based single index fiber is in medical diagnosis. Infrared spectroscopy is a well-adapted technique permitting the characterization of complex substances like proteins, nucleic acids, and lipids which are the main constituents of biological systems, so, it possesses aptitudes for characterizing precocious metabolic anomalies in different pathologic environments. In the future, thanks to the inertia of Se-based glass fibers towards biological substances [27] and their length and flexibility, the fibers could be implemented directly on patients by guiding the probe light onto the area of interest, rather than performing biopsies.

For medical diagnosis, for example, FEWS using glass fibers could analysis metabolic abnormalities by placing 10 µL of serum in contact with a fiber. By adequate statistical analyses, a principal component analysis (PCA) map, which could provide spectral fingerprints containing the information necessary to discriminate between ill and healthy patients, could be formed. In PCA maps, each spectrum is represented by a point in a two or three dimensional space.

**Figure 3:**
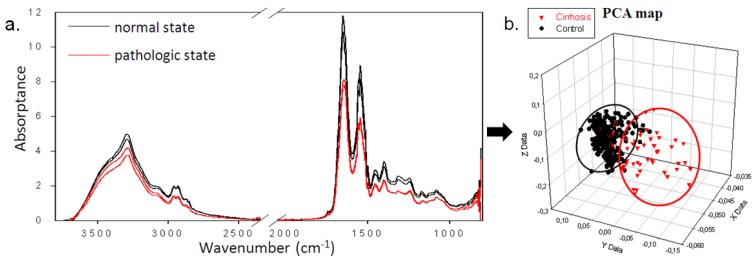
(**a**) Traditional optical absorptance spectrum of human serum with and without cirrhosis; (**b**) The principal component analysis (PCA) map got by statistical analysis.

In 2004, Keirsse [5] reported the first study on sera from mice developing obesity related to a homozygous mutation in the leptin gene, leading to hyperphagia and type II diabetes. The fiber evanescent wave spectra of these sera were compared with the spectra of mice that do not develop obesity as control spectra. All spectra were processed by PCA in the 1,100–1,000 cm^−1^ range, which corresponds to the sugar ring vibration bands. On the PCA map, the points corresponding to control spectra are well grouped and the spectra for obese mice are localized in a totally different area, spread over a larger zone, so this experiment proved that the FEWS technique coupled with an analysis method like PCA is an efficient tool for distinguishing pathological sera from normal ones. Figure 3 shows a comparison between traditional optical absorptance spectra and PCA signal after statistical analyses of human serum with and without cirrhosis. It can be seen that classical analysis methods are not able to differentiate between both families of spectra, but the two different metabolic states can be easily separated in a PCA map. These successful results have given rise to the founding of a startup company called DIAFIR [28], which aims at developing a global method of IR sensing including Se-based glass optical fibers and statistical analysis method into the chain of measurements. 

Moreover, in the biomedical area, Se-based fiber for FEWS could also been utilized in analysis of tissues [29] and monitoring of bacterial biofilms [27]. By using TAS glass fiber [23], the protein IR signatures of healthy tissues in the case of mouse liver were detected, indicating a high possibility of the substitution of traditional method through a CaF_2_ window.

FEWS can also be utilized in chemical reaction monitoring, such as in fermentation processes [12] and resin polymerization [20]. Besides, detection of pollutants in water [11] is also achievable. Finally, this setup has also given interesting results for the monitoring of gaseous CO_2_ [26]. In order to detect and monitor CO_2_, some more sophisticated rare-earth doped fibers have been recently developed. They are used as secondary sources excited by a laser pump working in the visible range.

## 3. Rare-Earth Doped Selenide Glass Fibers for IR Sources

Sources and lasers in the mid-infrared (mid-IR) are essential for a variety of applications, including environmental sensing, LIDAR and military counter-measures. However, coherent and incoherent sources, which are powerful, robust and compact features, are not available in this wavelength range. As many rare-earth (RE) elements show their capability in mid-IR emission, for potential applications in this spectral range, a strong RE mid-IR emission requires the use of material host with low phonon energy as sulfide (~350 cm^−1^) and selenide (~250 cm^−1^) matrices, for instance [30,31]. With high refractive index values and appropriate RE solubility, chalcogenide glasses exhibit high spontaneous emission probabilities and, consequently, large emission cross-sections of radiative electronic transitions of RE^3+^ ions. Besides, the low phonon energy of these glasses limits the non-radiative multiphonon relaxation rates. All these properties result in high quantum efficiencies for RE ion energy transitions in chalcogenide glasses [32,33,34,35,36,37,38]. Many radiative transitions have been recorded in the near and mid-IR spectral range in bulk chalcogenide glasses, mainly doped with Pr^3+^, Tb^3+^, Dy^3+^, Nd^3+^, Ho^3+^, Er^3+^, and Tm^3+^ ions. However, infrared emissions from rare earth ion-doped amorphous chalcogenide fibers are more rarely reported [32,34,36,38,39]. 

As Ga-Ge-Sb-Se glass presents a suitable RE^3+^ ion solubility and appropriate thermo-mechanical properties for optical fiber drawing [34,40] the Dy^3+^ and Pr^3+^-doped Ga_5_Ge_25_Sb_10_Se_60_ glasses and fibers with RE concentration varying from 0.05 up to 0.1 wt.% were explored according to the high quantum efficiencies in the 4–5 μm spectral domain of selenide glasses for developing remote optical sensor based on mid-IR sources [41]. These glasses were prepared by means of conventional melting and quenching methods [38,42,43,44]. Single refractive index fibers with 300–400 µm diameters were obtained for which Dy^3+^ or Pr^3+^ doping concentration was limited to 0.1 wt.%. This low concentration can avoid ion-ion dipole interactions and preserve classical optical losses of RE doped selenide fiber (1–3 dB/m). The rare earth spectroscopic study was carried out on both bulk glasses and fibers and is summarized in this article.

### 3.1. Dy^3+^ -Doped Glasses and Fibers

**Figure 4:**
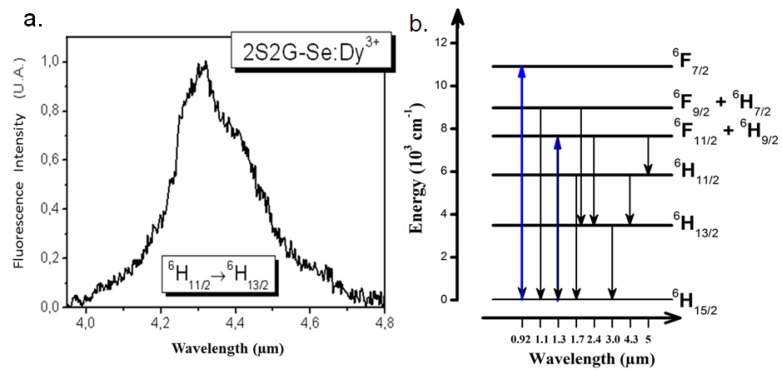
(**a**) Room-temperature fluorescence spectrum of Dy^3+^:Ga_5_Ge_25_Sb_10_Se_60_ bulk glass excited at 920nm; (**b**) The energy diagram of Dy^3+^ ion in Ga_5_Ge_25_Sb matrix.

For Dy^3+^ doped Ga_5_Ge_25_Sb_10_Se_60_ glass, the transmission limit at lower wavelengths was located at about 860 nm. Absorption spectrum of bulk glass showed five absorption bands around 2,865, 1,710, 1,305, 1,110 and 915 nm, corresponding to transitions from the ground state ^6^H_15/2_ (Figure 4b). The absorption cross-section of GaGeSbSe glass is greater than the GaGeSbS sulfide glass, for instance the transition ^6^H_15/2_°^6^F_11/2_+^6^H_9/2_ presents 4.6 × 10^−20^ cm^2^ compared to 3.5 × 10^−20^ cm^2^. Considering their absorption cross-section and wavelength cut-off, 920 nm (Ti:sapphire laser) and 1,300 nm (OPO pumped at 832 nm by Ti: sapphire laser) appear to be the best choice to pump Dy^3+^ ions in glasses and fibers, respectively. After Dy^3+^ ions were optically pumped to the ^6^H_11/2_ or an upper level, both the bulk glass and glassy fiber show a mid-IR emission at 4.35 µm with a FWHM of about 150 nm (Figure 4a), corresponding to the transition from ^6^H_11/2_ to ^6^H_13/2_.

Meanwhile, fluorescence decays were measured for Dy^3+^-doped Ga_5_Ge_25_Sb_10_Se_60_ splinters using a Nd:YAG-pumped OPO laser . The lifetimes were obtained by resonant excitations except for ^6^H_13/2_. In the case of ^6^H_13/2_ level, the lifetime recorded at 3 μm was obtained by excitation at 1.3 microns (^6^H_15/2_° ^6^F_11/2_+^6^H_9/2_). The decay lifetime is 3.4 ± 0.1ms for ^6^H_13/2_, 1.18 ± 0.05 ms for ^6^H_11/2_, 0.20 ± 0.01 ms for ^6^F_11/2_ + ^6^H_9/2_ and 0.05 ± 0.01 ms for ^6^F_9/2_ + ^6^H_7/2_. These values are in agreement with literature related to Dy^3+^ chalcogenide glasses [31,45]. Fluorescence quenching occurs with increasing Dy^3+^ concentration indicating non-radiative interactions among RE ions. A Dy^3+^ concentration of 0.05 wt.% was found to be suitable to limit reabsorption and energy transfer processes and was selected for fiber fabrication in order to improve emission efficiency. A cascade lasing associating the ^6^H_11/2_°^6^H_13/2_ transition with the ^6^H_13/2_^6^H_15/2_ transition was proposed to depopulate the ^6^H_13/2_ level presenting a long lifetime to prevent a blockage of the laser system [35]. The Dy^3+^:Ga_5_Ge_25_Sb_10_Se_60_ fiber is still attractive system for developing mid-IR sources. 

### 3.2. Pr^3+^ -Doped Glasses and Fibers

**Figure 5:**
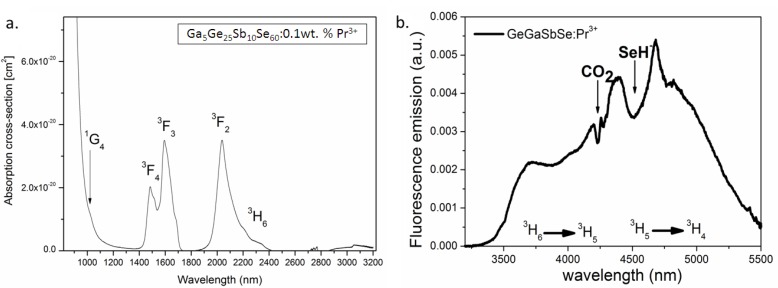
(**a**) Absorption cross-coefficient of Pr^3+^: GaGeSbSe glass; (**b**) Fluorescence emission of Pr^3+^:GaGeSbSe fiber excited at 2 µm. Such fibers, emitting in the mid-IR are very promising in view of detecting chemical species absorbing in the 3.5 to 5.5 µm range.

For Pr^3+^ doped Ga_5_Ge_25_Sb_10_Se_60_, the absorption measurements show two absorption bands and one shoulder at 2, 1.5 and 1 µm, corresponding to the transitions from the ^3^H_4_ ground state to (^3^H_6_, ^3^F_2_), (^3^F_4_, ^3^F_3_), and ^1^G_4_ states, respectively (Figure 5a). The (^3^F_4_, ^3^F_3_), and (^3^H_6_, ^3^F_2_) levels can be pumped by commercial diode lasers or silica-based fiber lasers operating at 1.5 and 2 μm, respectively. A homemade Tm: YAG laser at around 2 µm was used to pump Pr^3+^ in both Ga-Ge-Sb-Se fibers and bulk glasses to ^3^F_2_ level. The emission observed in mid-IR is very broad (3.5–5.5 µm), composed of ^3^H_6_°^3^H_5_ and ^3^H_5_°^3^H_4_ transitions (Figure 5b). The ratio between these two transitions depends on the matrix and on the reabsorption due to the ^3^H_4_°^3^H_5_ transition. 

Even if the concentration remains lower than 100 ppm after the purification process, impurities like Se-H (in Ga-Ge-Sb-Se) in chalcogenide glass can cause non-radiative relaxation due to their quasi-resonant phonon energy with mid-IR transitions. It is therefore necessary to further improve purity of glasses enabling a fluorescence signal as wide and flat as possible. From Figure 4b, it could also be noticed that the CO_2_ in air showed an obvious absorption peak located at around 4.3 μm even if the concentration is as small as 391 ppm. Consequently a fluorescent source based on Pr^3+^ doped Ga_5_Ge_25_Sb_10_Se_60_ fiber is surely appropriate for sensing molecules whose vibration absorption is located in the broad IR-emission band of Pr^3+^ from 3.5 to 5.5 μm.

For RE-doped Ga_5_Ge_25_Sb_10_Se_60_ glasses, although it is necessary to further improve emission intensity and explore new RE-doped glass systems enabling a fluorescence signal as wide and flat as possible, the results obtained on Dy^3+^ or Pr^3+^:Ge-Ga-Sb-Se glass system are very promising for future applications in the creation of mid-IR sources.

## 4. Selenide Microstructured Optical Fibers

**Figure 6:**
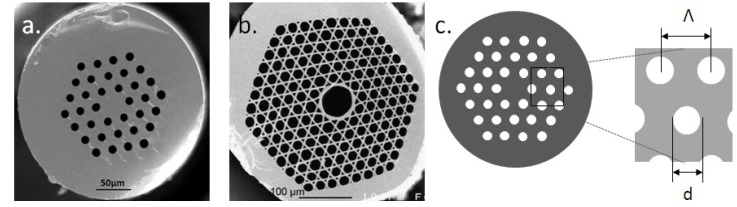
Transverse section of (**a**) Modified total internal reflection microstructured optical fibers (MTIR-MOFs); (**b**) Photonic band gap microstructured optical fibers (PBG-MOFs) [6] and (**c**) schematic diagram of hole diameter, d, hole spacing, Λ.

Microstructured optical fiber (MOF) is an optical fiber waveguide where guiding is achieved through manipulation of waveguide structure rather than its index of refraction. There are two main classes of MOF have been widely studied: solid-core MOFs (Figure 6a) and hollow core MOFs (Figure 6b). Except for Bragg fibers, which are composed of successive circular layers presenting two different refractive indices, both kinds of MOFs present periodic arrays of cylindrical air holes running along their entire length that corresponds to a 2D photonic crystal.

In the case of solid-core fibers, the refractive index of the center is higher than the effective index of the surrounding array composed of inclusion of lower refractive indexes. This is quite similar to a classical step-index fiber, guided by total internal refection. Thus, this kind of fibers is called modified total internal reflection microstructured optical fibers (MTIR-MOFs). For hollow core MOFs, the periodic structure causes a real photonic band-gap effect, as observed for example for the color of the butterfly wings. Therefore, these fibers are called photonic band gap microstructured optical fibers (PBG-MOFs).

In any case, for PBG-MOFs or MTIR-MOFs, the optical properties of the fibers are closely related to geometry: core size, number of rings, hole diameter, d, hole spacing, Λ, and also the ration d/Λ (Figure 6). Numbers of guided modes, mode effective area, broadband-single mode guidance, nonlinearity, band-gap width, and dispersion are examples of properties that can be managed by the geometry of MOFs.

In 2006, light guidance and the demonstration of single-mode propagation of sulfur based MOFs was successively performed [46,47]. Then we started to elaborate Se-based MOFs in 2008 including MTIR-MOF [48] and PBG-MOF [6]. These first achievements were elaborated using a “stack and draw” technique. To get a preform by this method, chalcogenide glass capillaries are stacked in a hexagonal lattice around a glass rod of an identical diameter and introduced in a jacket tube. It has been shown that defects such as bubbles appear at the glass interfaces and induce strong optical losses above 20 dB/m, therefore, the interstitial holes should remain open to improve the transmission of the glass fiber. Then the attenuation can be reduced to a few dB/m.

However, compared to the attenuation of classic single-index fibers, an excess of losses is always observed. In order to reduce optical losses, molding methods, which use structured silica array to mold glass, were developed. After a glassy liquid totally fills the mold, a preform could be formed by a traditional quenching-annealing method of this ampoule. The silica capillaries could be removed by hydrofluoric acid. The implementation of this molding technique avoids the occurrence of strong additional optical losses, and allows the fabrication of arsenic selenide MOFs with attenuations lower than 1 dB/m, which is close to the material ones [49,50].

**Figure 7:**
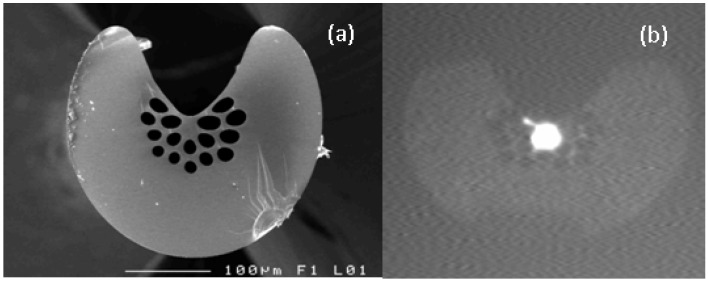
AsSe based microstructured exposed core fiber: (**a**) SEM micrographs of the. Fiber, external diameter d = 290 μm, core diameter d_c_ = 15 μm. (**b**) Near field capture at 1.55 µm showing the light confinement in the exposed core.

Generally speaking, in the frame of sensing applications, such Se-based MOFs are especially suitable to trap and monitor gaseous samples. Thus, it has been shown that the infrared signature of gases embedded in the holes of chalcogenide MTIR-MOFs can be detected. Indeed, by using arsenic selenide (As_40_Se_60_) glass MOFs, the absorption peak of CO_2_ gas at 4.2 μm has been successfully observed [51] by recording the transmittance spectrum when the fiber is filled with a flow of 100% carbon dioxide gas. Besides, in order to permit sufficient optical interactions between the core of the fiber and the chemical species, an exposed-core chalcogenide MOF has been demonstrated (Figure 7) for the first time [7]. Although evanescent wave absorption is inversely proportional to the fiber diameter, the result shows than an exposed-core fiber is much more sensitive than a single-index fiber with a twice-smaller external diameter.

On the other hand, it has been shown that chalcogenide MOFs with a large number of air-hole rings can even be endlessly single more when the ratio d = Λ is under 0.42. As an example, single-mode behavior has been observed in a Te_20_As_30_Se_50_ MTIR-MOF presenting three rings of holes [52]. In 2012, Ge_10_As_22_Se_68_ endlessly single-mode MOF was obtained [8] with optical losses equal to 1 dB/m at 1.55 µm. As explained in the next section, such single mode devices are essential to carry out interferometry experiments working in the mid-IR. Moreover, these MOF which combine the high non-linearity of the chalcogenide glasses with their holey design are especially suitable to generate broad band supercontinuum sources [53,54]. In the future, these fibers could be used as remote sources for infrared spectroscopy following the strategy exposed for the rare earth doped fibers in the paragraph above.

## 5. Single Mode Selenide Glass Fibers for Spatial Application

In recent years, space programs whose goal is to detect extra solar terrestrial planets have emerged. Indeed, the Darwin project from European Space Agency (ESA) and the “Terrestrial Planet Finder” project of the National Aeronautics and Space Administration (NASA) are two research programs whose goals are to find exoplanets having the same conditions as Earth and able to sustain life as we know it. The chemical signatures of life are exemplified by the presence of water, ozone and carbon dioxide in the planet atmosphere, with their absorption peaks located at 6.2, 9 and 15 µm, respectively. To demonstrate the presence of theses IR signatures, the strong-emittance from a parent star should be efficiently filtered using a technique called nulling interferometry. To fulfill the severe requirements, a flotilla of telescopes positioned with high precision and IR single-mode fibers are needed. ESA has defined a two wavelengths band system. The short wavelength for 6–12 μm is designed for H_2_O and O_3_ detection and long wavelength covering 12–20 µm is just suitable for CO_2_ detection. Therefore, single mode selenide glass fibers had to be developed permitting the propagation and selection of light existing in the short wavelength window.

**Figure 8:**
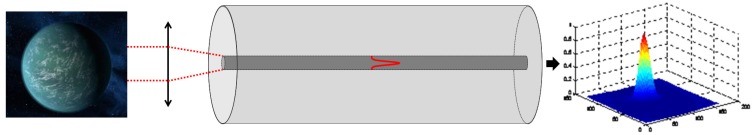
Optical configuration of a single mode, core/clad fiber required for space exploration by filtering the signal in the mid-IR.

In 2007, a step-index Te_20_As_30_Se_50_ glass fiber was developed [13] which showed single mode propagation at 10.6 μm with an adequate coupling efficiency. Two fibers, both with a core diameter of 11 μm and a clad diameter of 250 μm, were prepared by the classical rod in tube method and internal built-in-casting method. Both fibers exhibited a cut-off wavelength of 3.7 μm and an optical loss less than 0.1 dB/cm at 10.6 μm. These single mode TAS fibers could be considered as a first step towards the realization of the requirement of Darwin mission. The typical optical configuration of a single mode fiber and the experimental result obtained are shown in Figure 8.

For the wavelengths beyond 12 μm, involving the CO_2_ absorption band at around 15 μm, it will be necessary to use other materials with lower phonon energy. Promising materials are new glasses based exclusively on tellurium.

## 6. Telluride Glass Fiber for Far Infrared Experiments

Although Se and Te have the same hexagonal structure, it is well known that, in contrast to Se, which is a good glass-forming element, Te is impossible to vitrify, even when using fast quenching. Indeed, the Te melt, which is very fluid, possesses a strong metallic character. However, in order to successfully capture far infrared signals such as the CO_2_ absorption peak located at 15 μm for environmental applications and the Darwin mission, Te-based glass fibers should be developed. This asks for glasses which are stable enough against crystallization to allow the manufacturing of optical fiber.

Since 2006, our lab started to explore new materials containing large amounts of tellurium for CO_2_ detection [14]. Since then, new materials containing large amounts of tellurium have been developed in the Te-Ge-Ga [14] and Te-Ge-I [15] systems. 

The Te-Ge-Ga ternary system containing 70 to 80% of Te was the first Te-based glass composition reported for bulk and fiber optics. These bulk glasses present an exceptional optical-transmission window, lying between 2 and 28 μm [14]. The lowest optical loss is close to 0.6 dB/cm in the wavelength range of 6–20 μm [17]. However, as the maximum ΔT is limited, this composition shows a high tendency to crystallize during the fiber drawing process. Hence, Te-Ge-I [15] system was developed since 2007. The optimally stable glass was found to be Ge_20_Te_73_I_7_ with a ΔT value of 124 °C. However, as the glass forming zone of this ternary system is very narrow, a small deviation from the ideal composition due to the volatility of iodine during synthesis could lead to rapid loss of stability of the glassy materials. Therefore, for double index fiber, it is extremely tricky to control the composition deviations between core and clad to get single mode propagation.

In order to overcome those issues, more recently, the Te-Ge-Se [55,56,57] and Te-Ge-AgI [16] systems exhibiting broad infrared transmission and superior stability have been explored. These recent glassy systems open the way toward the fabrication of single mode optical fibers. This work is ongoing and such an achievement has yet to be made.

Additionally, such single index telluride optical fibers will also be very useful in the frame of medical applications. Indeed, they give access to infrared signal located further at wavelengths that will provide fruitful information which are not accessible with selenide glass fibers. The use of telluride glass fiber in that context is also in progress.

## 7. Conclusions

During the last decade, both selenide and telluride glassy optical fibers have shown their potential for mid-IR sensing in many fields of application. Selenide glasses are stable enough to give rise to sophisticated optical guiding devices such as single mode fibers, MOF or RE doped fibers. Each transduction mode is specific to each kind of fiber, which presents its own advantage as infrared sensing system: optical filtering for space, gaseous monitoring, *in-situ* motoring, *etc*. At this time, the most exciting application which is also the most mature, concerns the development of tapered mono-index selenide fibers used for medical diagnosis [58]. This technology is at the origin of the funding of a company [28]. On the other hand, telluride glass fibers become essential to probe the far-infrared, beyond 12 µm.
